# Normalized Brain Tissue–Level Evaluation of Volumetric Changes of Youth Athletes Participating in Collision Sports

**DOI:** 10.1089/neur.2021.0060

**Published:** 2022-01-28

**Authors:** Pratik Kashyap, Trey E. Shenk, Diana O. Svaldi, Roy J. Lycke, Taylor A. Lee, Gregory G. Tamer, Eric A. Nauman, Thomas M. Talavage

**Affiliations:** ^1^ Department of Electrical and Computer Engineering, Purdue University, West Lafayette, Indiana, USA.; ^2^ Weldon School of Biomedical Engineering, Purdue University, West Lafayette, Indiana, USA.; ^3^ School of Mechanical Engineering, Purdue University, West Lafayette, Indiana, USA.; ^4^ Department of Biomedical Engineering, University of Cincinnati, Cincinnati, Ohio, USA.

**Keywords:** cerebrospinal fluid, gray matter, head acceleration exposure, T1-weighted magnetic resonance imaging, tissue volumetry, youth athletes

## Abstract

Observations of short-term changes in the neural health of youth athletes participating in collision sports (e.g., football and soccer) have highlighted a need to explore potential structural alterations in brain tissue volumes for these persons. Studies have shown biochemical, vascular, functional connectivity, and white matter diffusivity changes in the brain physiology of these athletes that are strongly correlated with repetitive head acceleration exposure. Here, research is presented that highlights regional anatomical volumetric measures that change longitudinally with accrued subconcussive trauma. A novel pipeline is introduced that provides simplified data analysis on standard-space template to quantify group-level longitudinal volumetric changes within these populations. For both sports, results highlight incremental relative regional volumetric changes in the subcortical cerebrospinal fluid that are strongly correlated with head exposure events greater than a 50-G threshold at the short-term post-season assessment. Moreover, longitudinal regional gray matter volumes are observed to decrease with time, only returning to baseline/pre-participation levels after sufficient (5–6 months) rest from collision-based exposure. These temporal structural volumetric alterations are significantly different from normal aging observed in sex- and age-matched controls participating in non-collision sports. Future work involves modeling repetitive head exposure thresholds with multi-modal image analysis and understanding the underlying physiological reason. A possible pathophysiological pathway is presented, highlighting the probable metabolic regulatory mechanisms. Continual participation in collision-based activities may represent a risk wherein recovery cannot occur. Even when present, the degree of the eventual recovery remains to be explored, but has strong implications for the well-being of collision-sport participants.

## Introduction

Traumatic brain injury (TBI) records 1.6 million to 3.8 million cases annually.^1^ Of these numbers, mild TBI (mTBI) represents ∼80–90% of cases.^2^ Long-term risks involved with mTBI are neurological disorders, such as mild cognitive impairment and Alzheimer's disease^3^; psychiatric disorders, such as depression and post-traumatic stress disorder^4^; and, in certain cases, may include chronic traumatic encephalopathy (CTE).^5–7^

Sports-related mTBI is documented in athletes playing collision sports, such as football and soccer.^8^ It should be noted that 50–90% of sports-related mTBIs go undiagnosed,^9,10^ which raises serious concerns for athlete well-being. Football and soccer account for most high school sports-related mTBI.^11,12^ Concerns for the neural health of youth athletes are further highlighted by the observation of changes in neurological behavior without clinically observed impairment.^8^ These changes can be explained by asymptomatic (“subconcussive”) head acceleration events (HAEs) that cause silent cognitive deficits or other neurophysiological impairment.^13^

Structural health monitoring with multi-modal assessment of effects from repetitive HAEs^12,14^ points to mechanical stress as a likely cause of asymptomatic injury. Neurological changes have been evidenced biochemically,^15^ through modulation of vascular autoregulatory capacity,^16^ by alternations in visual working memory,^17,18^ by changes in functional network connectivity,^19–21^ and altered measures of white matter diffusivity.^22–26^ All these measures have been found to be linked to kinematic correlates obtained from on-field telemetry.^13,14,27^

Given the prevalence of neurophysiological changes with exposure to repetitive HAEs, it is reasonable to assess whether these alterations are associated with longitudinal structural volumetric changes. Existing methods to make such a determination include automated voxel-based morphometry (VBM), using tools such as FSL-SIENA,^28^ FSL-VBM,^29–32^ model-based FSL-FIRST,^33^ FreeSurfer-MorphometryStats,^34^ and technician-based, manually segmented regional volumetry techniques^35,36^ (ITK-SNAP v3.8^37^).

Here, a region-based volumetric analysis was conducted on a data set of high-school collision sport athletes (CSAs) known to have experienced repetitive HAEs. Past work has revealed longitudinal region-specific volumetric changes in both gray matter (GM)^38,39^ thickness, ventricular cerebrospinal fluid (CSF) increase,^40–42^ and white matter (WM) volumetry^43^ in subjects with mTBI. Volumetric measures were evaluated in collision-sport athletes (male football, female soccer) and non-collision-sport peers, including whole-brain WM,^44^ ventricular CSF (dCSF),^45^ and GM^46^ parcellations. Observation of longitudinal changes in regional tissue volumes that are linked to exposure to repetitive HAEs should provide insight as to how accumulation of mechanical strain may produce a metabolic cascade that has potential for longer-term neural health.

## Methods

### Participants

Eighty-six high-school–aged athletes participated on a voluntary basis, comprising two pools: 57 athletes participating in CSA and 29 athletes participating in non-collision sports (NCA; e.g., track and field, gymnastics, and cross-country). Participants were not excluded from either pool if they had a history of concussion. Participant demographics are provided in Table 1.

**Table 1.** Demographics of Participants with a Complete Set of Valid Imaging Data as Collected at *Pre/Test* Session

	Males		Females	
Category	CSA	NCA	CSA	NCA
	(*N* = 38)	(*N* = 15)	(*N* = 19)	(*N* = 14)
Age (years)	16.6 ± 1.2	16.2 ± 1.1	15.7 ± 1.2	15.8 ± 1.2
	[15, 18]	[15, 18]	[14, 18]	[14, 18]
Sport	Football (38)	Basketball (2)	Soccer (19)	Basketball (4)
		Track and field (4)		Track and field (3)
		Cross-country (1)		Gymnastics (1)
		Swimming (4)		Swimming (2)
		More than one (4)		More than one (4)
Years of high school athletics participation	2.1 ± 0.9	1.77 ± 1.1	1.9 ± 1.0	1.49 ± 1.1
	[0, 3]	[0, 4]	[0, 3]	[0, 3]
No. of previously diagnosed concussions	0.61 ± 1.0	0.48 ± 0.9	0.67 ± 1.1	0.51 ± 1.0
	[0, 5]	[0, 2]	[0, 3]	[0, 3]
Height (inches)	71.2 ± 2.7	70.6 ± 2.3	64.5 ± 2.2	66.4 ± 2.1
	[65, 77]	[66, 75]	[61, 69]	[62, 71]
Weight (lbs)	186.2 ± 34.6	152.6 ± 24.8	136.3 ± 18.4	127.1 ± 15.9
	[125, 245]	[120, 190]	[106, 170]	[110, 195]
Racial and ethnic categories				
White	30	13	19	11
Black or African American	5	0	0	1
Hispanic or Latino	2	0	0	0
Asian	1	2	0	2

All values are: mean ± StdDev, [Min, Max]).

CSA, collision sport athletes; NCA, non-collision sport athletes.

#### Collision sport athletes

Thirty-eight male (ages, 15–18 years) and 19 female (ages, 14–18 years) athletes, each participating in collision-based sports (M, American football; F, soccer), were recruited from three local high schools over four seasons of play.

#### Non-collision sports

Fifteen male (ages, 15–18 years) and 14 female (ages, 14–18 years) athletes, each participating only in non-collision sports, were recruited from the same high schools as the CSA participants. This pool served as a control for reproducibility of measurements over time. The Purdue Institutional Review Board approved the study procedures, and participants provided written informed consent before completion of the protocol.

### Participant schedule

**FIG. 1.**
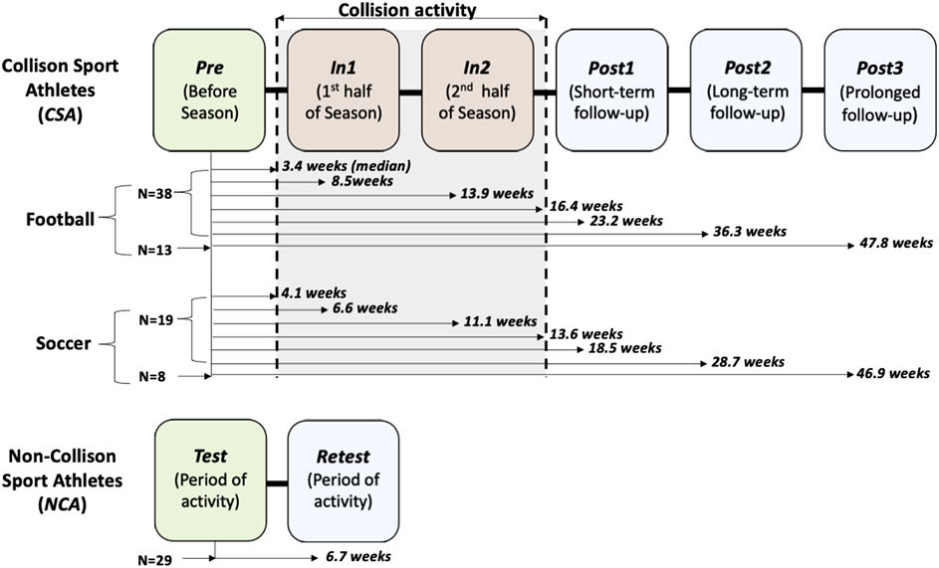
Schedule of longitudinal assessments for participants. Median intervals to each category of assessment are shown for each participant subgroup. (Top) Collision sport athletes (CSA) involved in boys' American football (*N* = 38) and girls' soccer (*N* = 19) were imaged at least five times around their competition season (*Pre*, during off-season conditioning; *In1*, during first half of the competition season [1–10 weeks after onset]; *In2*, during second half of the competition season [5–15 weeks after onset]; *Post1*, 1–2 months after end of the competition season [14–25 weeks after onset]; *Post2*, 4–6 months after end of the competition season [24–38 weeks after onset]; *Post3*, a subset of non-senior participants were imaged again 7–9 months after end of the competition season [44–52 weeks after onset]). (Bottom) Non-collision sport athletes (NCA; *N* = 29, F/M = 14/15) involved in non-collision sports (see Table 1) were imaged twice during competition and conditioning (*Test* and *Retest* separated by 1–2 months).

Imaging sessions and median intervals between sessions are schematized in Figure 1 for both CSA and NCA.

All CSAs underwent at least five magnetic resonance imaging (MRI) sessions around their competition season: *Pre*, before collision activity onset (i.e., before season-related activity commenced); *In1*, during the first half of the competition season; *In2*, during the second half of the competition season; *Post1*, near-term follow-up session after (1–10 weeks) competition season conclusion; and *Post2*, long-term follow-up session after (11–23 weeks) competition season conclusion.

For a subset of 21 CSA athletes, a sixth MRI session was conducted: *Post3*, prolonged follow-up session after (29–37 weeks) competition season conclusion. This imaging session was acquired as the *Pre* assessment for the subsequent season for persons who were not seniors at the time of *Pre*, *In1*, *In2*, *Post1*, and *Post2*. This session was only incorporated in analyses intended to examine the nature of volumetric changes after a prolonged period free of participation-related collision activity. Note that all CSAs were physically active, but not engaged in collision-related activities, before *Pre/Post3*, because they were all taking part in conditioning activities for their sports.

All NCAs underwent two MRI sessions, *Test* and *Retest*, separated in time by 4–13 weeks. All imaging sessions were conducted during training and competition activities to maintain comparable levels of physical activity around each session.

### Magnetic resonance imaging data acquisition

All imaging was conducted at the Purdue University MRI Facility (West Lafayette, IN), using a 3T General Electric Signa HDx (Waukesha, WI) with a 16-channel brain array (Nova Medical, Wilmington, MA). To assess the longitudinal stability of anatomical volumes, a high-resolution T1 acquisition (three-dimensional fast spoiled gradient-recalled echo; 1-mm isotropic resolution; repetition time/echo time = 5.71/1.976 msec; flip angle = 73 degrees) was conducted at each session.

### Head acceleration events data collection

CSAs were monitored for HAEs in each official practice and game. Telemetry data documenting peak translational acceleration (PTA) for each HAE were collected using one of two systems: the Head Impact Telemetry System (HITS; Simbex, Lebanon, NH), used only for American football athletes, or the xPatch (X2 Biosystems, Seattle, WA), used for both American football and soccer athletes. HITS encoders were used only with Riddell helmets, being placed before the season and examined thereafter only to recharge batteries or replace encoders that had ceased nominal operation. When the xPatch was used, the device was placed on the athlete's right ear using an adhesive patch before each practice or game and collected subsequent to the activity. Regardless of the system being used, a researcher was present at every session (practice or game) to ensure proper function of sensors, as previously described.^8,47^

Two CSAs were excluded from analysis because of incomplete HAE data: One football athlete ceased participation in the sport during the season; a second football athlete had his HITS encoder fail, and no replacement was available for several weeks.

### Magnetic resonance imaging data pre-processing

**FIG. 2.**
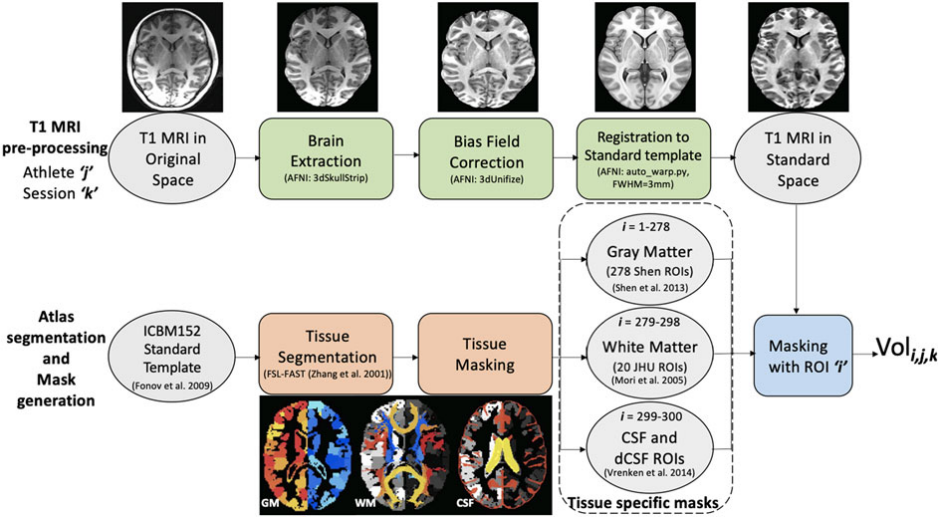
Pipelines for pre-processing, generation, and application of tissue-specific masks to T1w images for calculation of volumetric changes as a function of time. (Top) For each athlete (***j***) and session (***k***), the native-space T1w MRI was converted to the International Consortium of Brain Mapping standard space template/atlas (ICBM152)^48^ for subsequent masking. (Bottom) Three tissue-specific masks were generated and subsequently parcellated into 300 total regions of interest (ROIs; ***i***) for application to the standard space T1w images, allowing quantification of volumetric measurements in each region for each athlete and session (***Vol_i,j,k_***). Segmented tissues include gray matter (*GM*),^46^ white matter (*WM*),^44^ cerebrospinal fluid (*CSF*), and ventricular (or “deep”) cerebrospinal fluid (*dCSF*).^45^ Tools used: Analysis of Functional Neuroimages (AFNI)^49^ and the FMRIB software library (FSL).^31^ FWHM, full width at half maximum; JHU, Johns Hopkins University; MRI, magnetic resonance imaging; T1w, T1 weighted.

The top row of Figure 2 depicts the pre-processing pipeline conducted on each anatomical (T1-weighted [T1w] MRI) scan (i.e., for every athlete, at each session) to produce its representation in standard space, whereas the bottom row illustrates the segmentation and tissue mask generation of the standard space template, which is finally used to compute a volumetric measure for a segmented standard-space region of interest (ROI).

### Relative volume change computation

Relative volume changes were computed at three levels for each unique athlete session. First, the relative regional volume change (rRVC) was computed on an individual ROI basis, for each ROI associated with a given tissue type. Second, the relative tissue volume change (rTVC) was computed for each tissue type (GM, WM, CSF, and dCSF) by aggregating the relative volume changes in comprising ROIs. Third, relative brain volume change (rBVC) was computed by aggregating over relative tissue volume changes (GM, WM, and CSF) for the given athlete session.

To examine volumetric changes on a normalized region-specific basis, a novel pipeline was implemented. First, the rRVC in the *i^th^* ROI was computed for the *j^th^* athlete at the *k^th^* follow-up session (CSA, *In1*, *In2*, *Post1*, *Post2*, and *Post3*; NCA, *Retest*) with normalization to baseline (CSA, *Pre*; NCA, *Test*) volume as:

$$rRVC_{i,j,k}=\frac{Vol_{i,j,k}-Vol_{i,j,Pre}}{Vol_{i,j,Pre}}$$

The brain-wide measure of rTVC for a given athlete session was computed at each session by a weighted sum of the rRVCs obtained for each of the *N_X_* ROIs comprising tissue *X* ∈ {GM, WM, CSF, dCSF}:

$$rTVC_{X,j,k}=\sum_{i=1}^{N_{X}}c_{i}\times rRVC_{i,j,k}$$

The weight $c_{i}=\frac{v_{i}}{\sum_{k}v_{k}}$ is the relative fraction of the ROI volume (in voxels) represented by the ROI within the total volume of ROIs associated with the tissue.

$$rBVC_{j,k}=\sum_{X}w_{X}\times rTVC_{X,j,k}$$

The rBVC was similarly computed as the weighted sum of rTVC over the GM, WM, and CSF tissue masks, where *w_GM_* = 0.4262, *w_WM_* = 0.3912, and *w_CSF_* = 0.1826 represent the relative fraction of the entire brain encompassed by the GM, WM, and CSF masks, respectively, for the MNI-152 nonlinear sixth-generation atlas (ICBM152).^48^

### Head acceleration event processing

HAE data were pooled over the HITS and xPatch sensors and subsequently pruned by following the windowing and thresholding (≥20 G) procedure of McCuen and colleagues.^47^ Note that whereas both devices have been documented to exhibit low average error over large sample sizes,^50,51^ both devices are also known to exhibit appreciable error levels on an individual measurement basis.^50^ No analyses were performed on the basis of singular events.

The count of HAEs exceeding a PTA threshold (*PTA*_Th_∈{20G, 25G, 30G…95G}) for the *j^th^* athlete at the *k^th^* (*k* ∈ *{In1, In2, Post1}*) follow-up session was computed as:

$$nHAE_{Th,j,k}=\sum_{p=1}^{M_{k}}u\left(PTA_{p,j,k}-PTA_{Th}\right)$$

where *M_k_* represents the total number of HAEs experienced before the *k^th^* session, and $u\left(\right)$ is the unit/Heaviside step function.

### Statistical analysis

Analyses were performed in MATLAB (R2020a; The Mathworks, Inc., Natick, MA), using the statistical approaches described in Glantz and colleagues.^52^ Independence of test classes is implied when comparing rTVC from different populations (CSA, NCA) and separate sessions, but not for separate tissues, given that they are partitions of the complete brain.

Before conducting analyses across populations, the rTVC values for NCA athletes at *Retest* were checked for any sex effects using an unpaired two-sample *t*-test. Thereafter, CSA athlete measures of rTVC were evaluated for sphericity (Mauchly's W) and normality (one-sample Kolmogorov-Smirnov) before a one-way repeated-measures analysis of variance (rANOVA) was used to determine whether longitudinal differences in rTVC for CSA athletes were associated with session. *Post hoc t*-tests were conducted on a pair-wise basis within the CSA population to identify session-specific changes (paired *t*-test) and across the CSA and NCA to detect any population differences (unpaired *t*-test).

Assessment of the correlation of volumetric changes (rTVC) with HAE exposure (across multiple PTA thresholds) was effected by computation of Pearson's *r* at each session. A linear predictor was subsequently modeled for rTVC as a function of number of HAEs (nHAE) at each session, only for those PTA_Th_ for which a significant *r* was observed. All statistical results from these assessments were corrected for multiple comparisons using the Bonferroni correction.

Localization of region-specific longitudinal changes in rRVC was effected for CSAs on a confidence interval (CI) basis, using the NCA population as a reference. In this case, each ROI in the CSA was assessed on a tissue-level basis to determine whether the average rRVC for the given tissue exhibited a mean change that fell outside the 95% CI for the specific tissue (i.e., rTVC) for the NCA. Thus, two ROI maps were generated for each tissue in CSA, identifying those ROIs for which the average CSA rRVC changes were 1) more negative than the lower 95% CI bound of the rTVC for NCA or 2) more positive than the upper 95% CI bound of the rTVC for NCA. Note that the WM assessment did not reveal any within-season ROIs falling outside the 95% CI and was omitted from further analysis.

## Results

Measurements of rTVC were found to be reliable in the control (NCA) population. rTVC values were found to be spherical and normal and were pooled across sex because the *Test-Retest* evaluation of NCA (rTVC_Retest_) was not found to differ significantly. Additionally, it is to be noted that rTVC_Retest_ values were not different in mean from zero over the 4- to 13-week time scale.

Mean scores of rTVC for GM and dCSF exhibited significant session-wise differences within CSA and between CSA and NCA. Longitudinal assessment (rANOVA) of rTVC for the CSA population was found to be significant for GM (*F*_(3,168)_ = 2.91) and dCSF (*F*_(3,168)_ = 3.13). *Post hoc* Bonferroni corrected *t*-tests of rTVC within the CSA population and across the NCA-CSA populations are presented in Table 2.

**Table 2.** Comparisons, by Tissue Type, of rTVC as a Function of Group and Session

	Mean difference in rTVC (%)									
Tissue	CSA vs. CSA						CSA vs. NCA			
	In2 vs. In1	Post1 vs. In1	Post2 vs. In1	Post1 vs. In2	Post2 vs. In2	Post2 vs. Post1	In1 vs. Retest	In2 vs. Retest	Post1 vs. Retest	Post2 vs. Retest
GM	–0.91	–1.55	0.88	–0.64	1.79	**2.43^a^**	–0.89	–1.80	**–2.44^a^**	–0.01
CSF	0.42	1.86	0.01	1.44	–0.41	–1.85	0.78	1.20	**2.64^a^**	0.79
dCSF	1.34	**3.68^a^**	0.05	2.34	–1.29	**–3.63^a^**	–0.02	1.32	**3.66^a^**	0.03

^a^ Values in bold are statistically significant at *p* < 0.05 after Bonferroni correction.

For each tissue of gray matter (GM), cerebrospinal fluid (CSF), and deep (ventricular) CSF (dCSF), results are presented for both within-group analysis of the collision sport athletes (CSA) and for an across-group analysis of CSA with the non-collision sport athletes (NCA). To evaluate within-group differences for CSA, Bonferroni-corrected paired *t*-tests were conducted to compare the mean difference in relative tissue volumetric changes (rTVC) on a pair-wise basis between follow-up sessions (*In1*, *In2*, *Post1*, and *Post2*). To evaluate CSA relative to NCA, Bonferroni-corrected unpaired *t*-tests were conducted to compare the mean difference in rTVC on a pair-wise basis between the CSA follow-up session and NCA follow-up session (*Retest*).

**FIG. 3.**
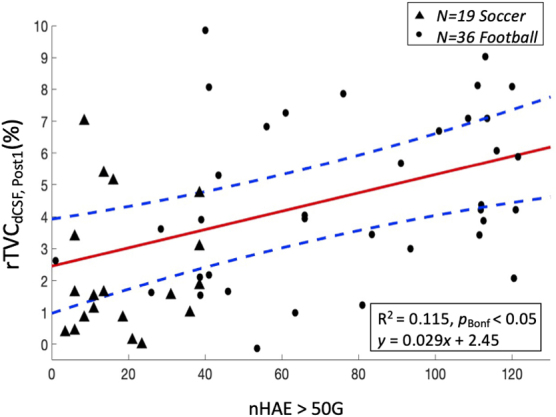
Linear dependence (*p_Bonf_* < 0.05) at *Post1* (*N* = 55) for percentage relative tissue volume change (rTVC) of ventricular cerebrospinal fluid (*dCSF*) as a function of the number of head acceleration events (nHAE) exceeding 50 G. Female soccer athletes (*N* = 19) and male football athletes (*N* = 36) are represented by solid triangles and solid circles, respectively. Red solid and blue dashed lines represent the mean regression line and corresponding 95% confidence interval. *Note*: Two football athletes with incomplete HAE data were excluded from this analysis.

Assessments of rTVC for dCSF demonstrated a linear relationship with accumulated repetitive head injury trauma (nHAE) at a PTA_Th_ of 50 G. The predictor modeling of rTVC as a function of nHAE for the significant PTA_Th_ session is illustrated in Figure 3 with respective 95% CIs.

Longitudinal volumetric measures of GM ROIs for CSA exhibited a concave up behavior. Values decreased from baseline (*Pre*) with maximum deviation at end of season, given that collision-based activity ceased (*Post1*), followed by a neuroplastic return to baseline (*Pre*) after an extended period of rest (*Post3*).

**FIG. 4.**
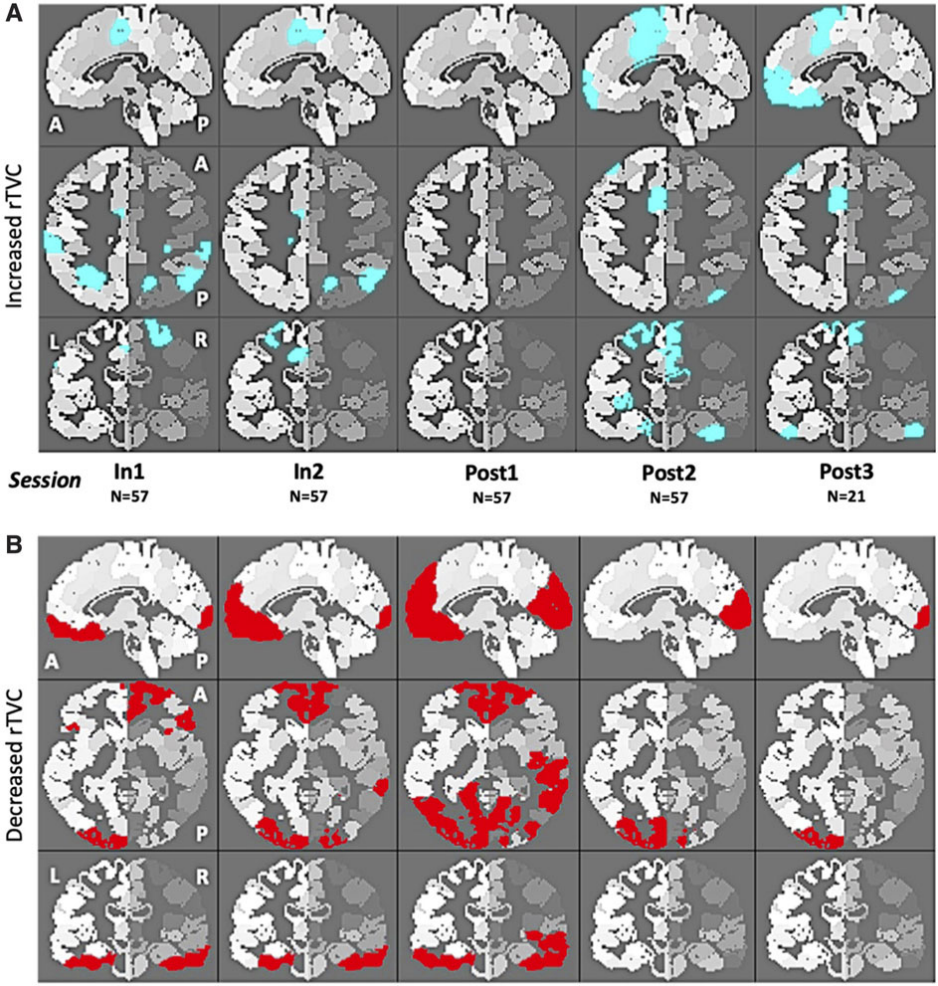
Gray matter regions of interest (ROIs)^46^ in collision sport athletes (CSA) at each given imaging session (see Fig. 1) who exhibited an average relative regional volume change (rRVC), relative to *Pre*, that fell outside the 95% confidence interval (CI) defined from *Test-Retest* evaluation of non-collision athletes (NCA; *N* = 29). (**A**) ROIs falling below the lower bound of the CI are depicted in cyan. (B) ROIs falling above the upper bound of the CI are depicted in red.

Figure 4A illustrates GM ROIs of GM for CSA exhibiting an average rRVC exceeding the upper 95% CI defined by rTVC of GM for NCA. The number of positively deviant ROIs peaked at *In1*, decreased with time until *Post1*, and then again increased across *Post2* and *Post3*.

Figure 4B illustrates GM ROIs for CSAs exhibiting an average rRVC falling below the lower 95% CI defined by rTVC of GM for NCAs. The number of negatively deviant ROIs increased longitudinally across sessions *In1-Post1*, but fell sharply for *Post2* and *Post3*.

## Discussion

This investigation characterized and explained variations in brain volume measures from repetitive mTBI experienced by the participation of high-school–aged youth in collision sports (CSA). Using MRI, volumetric changes were assessed across a season of collision-sport participation and compared with the session-to-session variability of age-matched peers who participate only in sports that do not involve purposeful collisions (NCA). Statistically significant rTVCs were observed for GM and dCSF in CSA at the post-season (*Post1*) measure, relative to measures obtained before the season's onset of collision exposure (*Pre*). Further, longitudinal volumetric dCSF increases in these athletes were found to be significantly correlated with exposure to repetitive mTBI (i.e., nHAE). Consistent with previous observations of changes in brain health in this population, the changes observed in dCSF did not return to baseline/normative levels until several months after the cessation of collision-based activities (*Post2*, *Post3*). Although persistent alterations such as those suggesting accrual of changes year over year were not observed, concern remains for the neural health of CSAs given the relatively long duration of the observed alterations in brain volume.

Documented volumetric changes for CSAs are suggestive of at least transient deleterious alterations to brain physiology brought about by repetitive HAEs. Decreases in GM volume and increases in dCSF volume are not consistent with previous literature quantifying year-to-year normative brain development.^53–55^ Further, volumetric changes in dCSF were linked to cumulative exposure to repetitive mTBI. Given the intersession intervals, and the subsequent neuroplastic return to baseline after extended rest from collision-based activity, it is unlikely that these observed changes are a consequence of maturation in adolescents. Note that we are here assuming that normative changes for youth athletes over the interval between imaging sessions are captured by our test-retest measures with the NCAs. The observation of significant tissue-level (GM, CSF, and dCSF) population-specific volumetric changes for CSA at *Post1* (Table 2) can be reasonably attributed to accumulated physiological changes caused by HAEs, which are expected to be absent (or at least substantially lesser) in NCA.

Near-term post-season measurements of volumetric changes in dCSF for asymptomatic CSAs were predicted by cumulative exposure to repetitive mTBI. The longitudinal tissue-level changes observed in mean rTVC for dCSF were strongly correlated at *Post1* with nHAE at a PTA threshold of 50 G (Fig. 3). Regional brain mechanical responses to randomly incident HAE exhibit a primary intersection of induced strain near the center of the brain.^56,57^ This accumulated strain might explain the observation of localized dCSF increase as opposed to a significant global change in CSF. These findings are not dependent on athlete sex even after considering differences in hit distribution profiles between football and soccer athletes.^58^ At this 50-G threshold, these volumetric findings complement previous detection of cerebrovascular reactivity (CVR)^16^ and magnetic resonance spectroscopy (MRS)^15^ changes for the asymptomatic CSA population. Further, similar PTA thresholds (e.g., 60 G) have been found to induce functional changes in previous biomarker studies involving this CSA population using MRS^59^ and working memory functional MRI (fMRI).^8,17^ These various findings combine to further our understanding of the threshold at which HAEs meaningfully affect brain health.

In contrast, regional variation of GM volumetric measures was sparsely scattered across all areas of the brain. Longitudinal measurements of volumetric changes in GM for CSA primarily showed an increase in parietal cortex (Fig. 4A) coupled with a decrease (Fig. 4B) in temporal, occipital, and pre-frontal cortex. At a regional level, similar longitudinal distributions of deviant ROIs in CSAs have been observed for task-based fMRI,^17^ CVR,^60^ diffusion tensor imaging (DTI),^24–26,61^ and neurophysiological impairment.^27^ Lack of consistent localization of these GM volumetric changes is likely attributable to player-specific patterns of mTBI history (location, magnitude, and frequency).^18,58^

Documenting and validating the loci of dCSF and GM volumetric changes should aid future investigations to explore regional alterations in brain physiology. Lack of volumetric changes in WM could be explained by the absence of immediate large-scale axonal loss,^62^ sensitivity of MRI as opposed to DTI to detect WM changes,^24^ and the relatively long duration for neuroinflammation to manifest as WM degeneration.^63^

Future intervention strategies for changes such as those that were here observed require us to explore possible mechanisms governing these volumetric changes. Longitudinal changes in volumetric measures of GM and dCSF (Table 2) reach a maximum 4–8 weeks after the cessation of collision activity and return to normal after 15–20 weeks of rest from collision-related activity. GM changes could be perfusion induced,^64–66^ with apoptosis of cortical cells peaking only after 1 week post-injury.^67,68^ dCSF increase could be attributed to improper waste metabolism within the glymphatic system,^69,70^ leading to a progressive enlargement of the ventricles. The cumulative effect of these asymptomatic volumetric changes could lead to the manifestation of a more gross and recognizable symptomatic pathology.

**FIG. 5.**
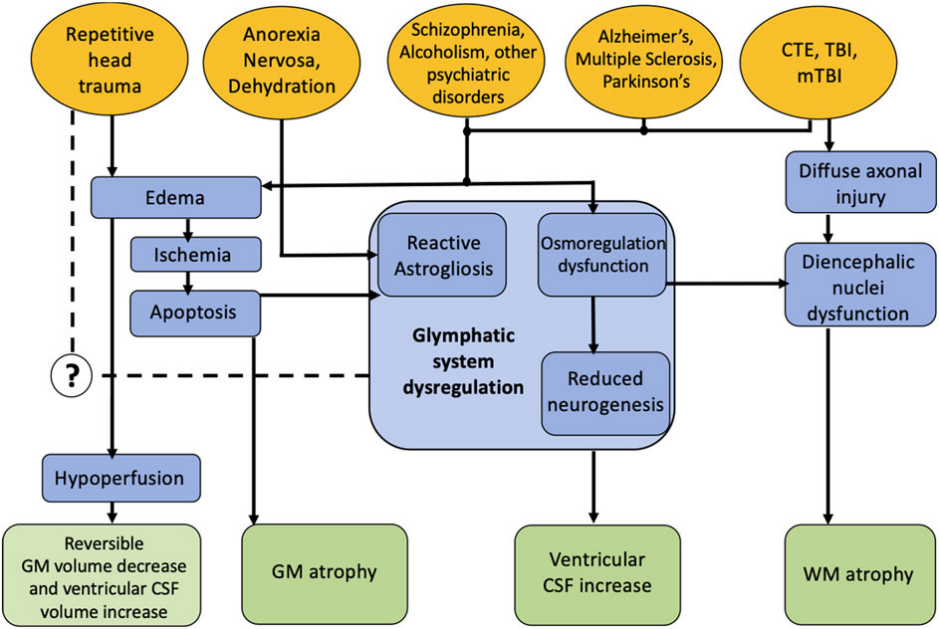
Hypothesis of pathophysiological mechanisms linking repetitive mTBI (as associated with exposure to HAEs) to observed reversible changes in *GM* and *dCSF* volumes. Other disorders (yellow) known to effect similar changes in *GM* and *dCSF*, in addition to changes in *WM* that were not observed herein, are depicted along with key pathways (blue) hypothesized to lead to these neurological observations (green). CSF, cerebrospinal fluid; CTE, chronic traumatic encephalopathy; dCSF, ventricular (or “deep”) cerebrospinal fluid; GM, gray matter; HAEs, head acceleration events; mTBI, mild TBI; TBI, traumatic brain injury; WM, white matter.

The exact mechanism of interaction of these tissue-specific volumetric changes and how they play a combined role in explaining asymptomatic repetitive HAE-induced injury remains yet to be quantified, but several hypothesized key pathways linking these neurological observations are presented in Figure 5. Pathologies such as anorexia nervosa^71–74^ and dehydration^75,76^ present with similar volumetric observations and therefore might narrow down the actively involved metabolic regulatory mechanisms. The acute edema, neuroinflammation,^24^ and oxidative stress^77^ from repetitive asymptomatic injuries could possibly lead to reactive astrogliosis,^78^ microglial activation,^79^ and alteration of astrocyte density and metabolism,^80–82^ coupled with reduced neurogenesis,^83–85^ mitochondrial dysfunction,^86,87^ and excitotoxicity.^88,89^ Efforts at harmonization of these mechanisms lead us to hypothesize a connection between glymphatic system dysregulation and repetitive mTBI. These mechanisms are possible precursors of large-scale systemic metabolic alterations, arising from accumulation of HAEs over longer periods of collision-based activity.

## Abbreviations Used

CI: confidence interval
CSAs: collision sport athletes
CSF: cerebrospinal fluid
CTE: chronic traumatic encephalopathy
CVR: cerebrovascular reactivity
dCSF: ventricular CSF
DTI: diffusion tensor imaging
fMRI: functional MRI
GM: gray matter
HAEs: head acceleration events
HITS: Head Impact Telemetry System
MRI: magnetic resonance imaging
MRS: magnetic resonance spectroscopy
mTBI: mild TBI
NCA: non-collision sports
nHAE: number of head acceleration events
PTA: peak translational acceleration
rANOVA: repeated-measures analysis of variance
rBVC: relative brain volume change
ROI: region of interest
rRVC: relative regional volume change
rTVC: relative tissue volumetric changes
T1w: T1-weighted
TBI: traumatic brain injury
VBM: voxel-based morphometry
WM: white matter

## References

[1] Langlois, J.A., Rutland-Brown, W., and Wald, M.M. (2006). The epidemiology and impact of traumatic brain injury: a brief overview. J. Head Trauma Rehabil. 21, 375–378. 16983222 10.1097/00001199-200609000-00001

[2] Bigler, E.D., and Maxwell, W.L. (2012). Neuropathology of mild traumatic brain injury: relationship to neuroimaging findings. Brain Imaging Behav. 6, 108–136. 22434552 10.1007/s11682-011-9145-0

[3] Guskiewicz, K.M., Marshall, S.W., Bailes, J., McCrea, M., Cantu, R.C., Randolph, C., and Jordan, B.D. (2005). Association between recurrent concussion and late-life cognitive impairment in retired professional football players. Neurosurgery 57, 719–726. 16239884 10.1093/neurosurgery/57.4.719

[4] Stein, D.J., Costa, D., Lochner, C., Miguel, E.C., Reddy, Y., Shavitt, R.G., van den Heuvel, O.A., and Simpson, H.B. (2019). Obsessive-compulsive disorder, Nat. Rev. Dis. Primers 5, 52. 10.1038/s41572-019-0102-3 PMC7370844 31371720

[5] Omalu, B.I., DeKosky, S.T., Minster, R.L., Kamboh, M.I., Hamilton, R.L., and Wecht, C.H. (2005). Chronic traumatic encephalopathy in a National Football League player. Neurosurgery 57, 128–134. 10.1227/01.neu.0000163407.92769.ed 15987548

[6] McKee, A.C., Cantu, R.C., Nowinski, C.J., Hedley-Whyte, E.T., Gavett, B.E., Budson, A.E., Santini, V.E., Lee, H.S., Kubilus, C.A., and Stern, R.A. (2009). Chronic traumatic encephalopathy in athletes: progressive tauopathy after repetitive head injury. J. Neuropathol. Exp. Neurol. 68, 709–735. 19535999 10.1097/NEN.0b013e3181a9d503 PMC2945234

[7] Changa, A.R., Vietrogoski, R.A., and Carmel, P.W. (2018). Dr Harrison Martland and the history of punch drunk syndrome. Brain 141, 318–321. 29325051 10.1093/brain/awx349

[8] Talavage, T.M., Nauman, E.A., Breedlove, E.L., Yoruk, U., Dye, A.E., Morigaki, K.E., Feuer, H., and Leverenz, L.J. (2014). Functionally-detected cognitive impairment in high school football players without clinically-diagnosed concussion. J. Neurotrauma 31, 327–338. 20883154 10.1089/neu.2010.1512 PMC3922228

[9] Baugh, C.M., Stamm, J.M., Riley, D.O., Gavett, B.E., Shenton, M.E., Lin, A., Nowinski, C.J., Cantu, R.C., McKee, A.C., and Stern, R.A. (2012). Chronic traumatic encephalopathy: neurodegeneration following repetitive concussive and subconcussive brain trauma. Brain Imaging Behav. 6, 244–254. 22552850 10.1007/s11682-012-9164-5

[10] Meehan, W.P. III, Mannix, R.C., O'Brien, M.J., and Collins, M.W. (2013). The prevalence of undiagnosed concussions in athletes. Clin. J. Sport Med. 23, 339–342. 23727697 10.1097/JSM.0b013e318291d3b3 PMC3758800

[11] Bailes, J.E., Petraglia, A.L., Omalu, B.I., Nauman, E., and Talavage, T. (2013). Role of subconcussion in repetitive mild traumatic brain injury. J. Neurosurg. 119, 1235–1245. 23971952 10.3171/2013.7.JNS121822

[12] Talavage, T.M., Nauman, E.A., and Leverenz, L.J. (2016). The role of medical imaging in the recharacterization of mild traumatic brain injury using youth sports as a laboratory. Front. Neurol. 6, 273. 26834695 10.3389/fneur.2015.00273 PMC4717183

[13] Nauman, E.A., Breedlove, K.M., Breedlove, E.L., Talavage, T.M., Robinson, M.E., and Leverenz, L.J. (2015). Post-season neurophysiological deficits assessed by ImPACT and fMRI in athletes competing in American football. Dev. Neuropsychol. 40, 85–91. 25961591 10.1080/87565641.2015.1016161

[14] Slobounov, S.M., Walter, A., Breiter, H.C., Zhu, D.C., Bai, X., Bream, T., Seidenberg, P., Mao, X., Johnson, B., and Talavage, T.M. (2017). The effect of repetitive subconcussive collisions on brain integrity in collegiate football players over a single football season: a multi-modal neuroimaging study. Neuroimage Clin. 14, 708–718. 28393012 10.1016/j.nicl.2017.03.006 PMC5377433

[15] Bari, S., Svaldi, D.O., Jang, I., Shenk, T.E., Poole, V.N., Lee, T., Dydak, U., Rispoli, J.V., Nauman, E.A., and Talavage, T.M. (2019). Dependence on subconcussive impacts of brain metabolism in collision sport athletes: an MR spectroscopic study. Brain Imaging Behav. 13, 735–749. 29802602 10.1007/s11682-018-9861-9

[16] Svaldi, D.O., Joshi, C., McCuen, E.C., Music, J.P., Hannemann, R., Leverenz, L.J., and Talavage, T.M. (2020). Accumulation of high magnitude acceleration events predicts cerebrovascular reactivity changes in female high school soccer athletes. Brain Imaging Behav. 14, 164–174. 30377933 10.1007/s11682-018-9983-0

[17] Arciniega, H., Shires, J., and Furlong, S. (2021). Impaired visual working memory and reduced connectivity in undergraduates with a history of mild traumatic brain injury. Sci. Rep. 11, 2789. 33531546 10.1038/s41598-021-80995-1 PMC7854733

[18] Shenk, T.E., Breedlove, E.L., Leverenz, L.J., Nauman, E.A., and Talavage, T.M. (2015). The role of location of subconcussive head impacts in fMRI brain activation change. Dev. Neuropsychol. 40, 74–79. 25961589 10.1080/87565641.2015.1012204

[19] Abbas, K., Shenk, T.E., Poole, V.N., Breedlove, E.L., Leverenz, L.J., Nauman, E.A., Talavage, T.M., and Robinson, M.E. (2015). Alteration of default mode network in high school football athletes due to repetitive subconcussive mild traumatic brain injury: a resting-state functional magnetic resonance imaging study. Brain Connect. 5, 91–101. 25242171 10.1089/brain.2014.0279

[20] Abbas, K., Shenk, T.E., Poole, V.N., Robinson, M.E., Leverenz, L.J., Nauman, E.A., and Talavage, T.M. (2015). Effects of repetitive sub-concussive brain injury on the functional connectivity of Default Mode Network in high school football athletes. Dev. Neuropsychol. 40, 51–56. 25649781 10.1080/87565641.2014.990455

[21] Johnson, B., Neuberger, T., Gay, M., Hallett, M., and Slobounov, S.M. (2014). Effects of subconcussive head trauma on the default mode network. J. Neurotrauma 31, 1907–1913. 25010992 10.1089/neu.2014.3415 PMC4238241

[22] Chun, I.Y., Mao, X., Breedlove, E.L., Leverenz, L.J., Nauman, E.A., and Talavage, T.M. (2015). DTI detection of longitudinal WM abnormalities due to accumulated head impacts. Dev. Neuropsychol. 40, 92–97. 25961592 10.1080/87565641.2015.1020945

[23] Bahrami, N., Sharma, D., Rosenthal, S., Davenport, E.M., Urban, J.E., Wagner, B., Jung, Y., Vaughan, C.G., Gioia, G.A., Stitzel, J.D., Whitlow, C.T., and Maldjian, J.A. (2016). Subconcussive head impact exposure and white matter tract changes over a single season of youth football. Radiology 281, 919–926. 27775478 10.1148/radiol.2016160564 PMC5131834

[24] Jang, I., Chun, I.Y., Brosch, J.R., Bari, S., Zou, Y., Cummiskey, B.R., Lee, T.A., Lycke, R.J., Poole, V.N., Shenk, T.E., Svaldi, D.O., Tamer, G.G. Jr., Dydak, U., Leverenz, L.J., Nauman, E.A., and Talavage, T.M. (2019). Every hit matters: white matter diffusivity changes in high school football athletes are correlated with repetitive head acceleration event exposure. Neuroimage Clin. 24, 1019–1030. 10.1016/j.nicl.2019.101930 PMC6807364 31630026

[25] Bazarian, J.J., Zhu, T., Zhong, J., Janigro, D., Rozen, E., and Roberts, A. (2014). Persistent, long-term cerebral white matter changes after sports-related repetitive head impacts. PLoS One 9, e94734. 24740265 10.1371/journal.pone.0094734 PMC3989251

[26] Lipton, M.L., Kim, N., Zimmerman, M.E., Kim, M., Stewart, W.F., Branch, C.A., and Lipton, R.B. (2013). Soccer heading is associated with white matter microstructural and cognitive abnormalities. Radiology 268, 850–857. 23757503 10.1148/radiol.13130545 PMC3750422

[27] Breedlove, E.L., Robinson, M., Talavage, T.M., Morigaki, K.E., Yoruk, U., O'Keefe, K., King, J., Leverenz, L.J., Gilger, J.W., and Nauman, E.A. (2012). Biomechanical correlates of symptomatic and asymptomatic neurophysiological impairment in high school football. J. Biomech. 45, 1265–1272. 22381736 10.1016/j.jbiomech.2012.01.034

[28] Smith S.M. (2002). Fast robust automated brain extraction. Hum. Brain Mapp. 17, 143–155. 12391568 10.1002/hbm.10062 PMC6871816

[29] Douaud, G., Smith, S., Jenkinson, M., Behrens, T., Johansen-Berg, H., Vickers, J., James, S., Voets, N., Watkins, K., Matthews, P.M., and James, A. (2007). Anatomically related grey and white matter abnormalities in adolescent-onset schizophrenia. Brain 130, Pt. 9, 2375–2386. 17698497 10.1093/brain/awm184

[30] Good, C.D., Johnsrude, I.S., Ashburner, J., Henson, R.N., Friston, K.J., and Frackowiak, R.S. (2001). A voxel-based morphometric study of ageing in 465 normal adult human brains. NeuroImage 14, 1 Pt. 1, 21–36. 11525331 10.1006/nimg.2001.0786

[31] Smith, S.M., Jenkinson, M., Woolrich, M.W., Beckmann, C.F., Behrens, T.E., Johansen-Berg, H., Bannister, P.R., De Luca, M., Drobnjak, I., Flitney, D.E., Niazy, R.K., Saunders, J., Vickers, J., Zhang, Y., De Stefano, N., Brady, J.M., and Matthews, P.M. (2004). Advances in functional and structural MR image analysis and implementation as FSL. NeuroImage 2,3 Suppl. 1, S208–S219. 10.1016/j.neuroimage.2004.07.051 15501092

[32] Andersson, M.J., and Smith, S.M. (2007). Non-linear registration, a.k.a. Spatial normalisation. FMRIB technical report TR07JA2. www.fmrib.ox.ac.uk/datasets/techrep/tr07ja2/tr07ja2.pdf (Last accessed January 10, 2022).

[33] Patenaude, B., Smith, S.M., Kennedy, D.N., and Jenkinson, M. (2011). A Bayesian model of shape and appearance for subcortical brain segmentation. NeuroImage 56, 907–922. 21352927 10.1016/j.neuroimage.2011.02.046 PMC3417233

[34] Buckner, R.L., Head, D., Parker, J., Fotenos, A.F., Marcus, D., Morris, J.C., and Snyder, A.Z. (2004). A unified approach for morphometric and functional data analysis in young, old, and demented adults using automated atlas-based head size normalization: reliability and validation against manual measurement of total intracranial volume. NeuroImage 23, 724–738. 15488422 10.1016/j.neuroimage.2004.06.018

[35] Hashempour, N., Tuulari, J.J., Merisaari, H., Lidauer, K., Luukkonen, I., Saunavaara, J., Parkkola, R., Lähdesmäki, T., Lehtola, S.J., Keskinen, M., Lewis, J.D., Scheinin, N.M., Karlsson, L., and Karlsson, H. (2019). A novel approach for manual segmentation of the amygdala and hippocampus in neonate MRI. Front. Neurosci. 13, 1025. 31616245 10.3389/fnins.2019.01025 PMC6768976

[36] Bartel, F., Vrenken, H., Bijma, F., Barkhof, F., van Herk, M., and de Munck, J.C. (2017). Regional analysis of volumes and reproducibilities of automatic and manual hippocampal segmentations. PLoS One 12, e0166785. 28182655 10.1371/journal.pone.0166785 PMC5300281

[37] Yushkevich, P.A., Piven, J., Hazlett, H.C., Smith, R.G., Ho, S., Gee, J.C., and Gerig, G. (2006). User-guided 3D active contour segmentation of anatomical structures: significantly improved efficiency and reliability. Neuroimage 31, 1116–1128. 16545965 10.1016/j.neuroimage.2006.01.015

[38] Bigler, E.D., Abildskov, T.J., Eggleston, B., Taylor, B.A., Tate, D.F., Petrie, J.A., Newsome, M.R., Scheibel, R.S., Levin, H., Walker, W.C., Goodrich-Hunsaker, N., Tustison, N.J., Stone, J.R., Mayer, A.R., Duncan, T.D., York, G.E., and Wilde, E.A. (2019). Structural neuroimaging in mild traumatic brain injury: a chronic effects of neurotrauma consortium study. Int. J. Methods Psychiatr. Res. 28, e1781. 31608535 10.1002/mpr.1781 PMC6877164

[39] Zhou, Y., Kierans, A., Kenul, D., Ge, Y., Rath, J., Reaume, J., Grossman, R.I., and Lui, Y.W. (2013). Mild traumatic brain injury: longitudinal regional brain volume changes. Radiology 267, 880–890. 23481161 10.1148/radiol.13122542 PMC3662902

[40] Brezova, V., Moen, K.G., Skandsen, T., Vik, A., Brewer, J.B., Salvesen, O., and Håberg, A.K. (2014). Prospective longitudinal MRI study of brain volumes and diffusion changes during the first year after moderate to severe traumatic brain injury. Neuroimage Clin. 5, 128–140. 25068105 10.1016/j.nicl.2014.03.012 PMC4110353

[41] Ledig, C., Kamnitsas, K., Koikkalainen, J., Posti, J.P., Takala, R., Katila, A., Frantzén, J., Ala-Seppälä, H., Kyllönen, A., Maanpää, H.R., Tallus, J., Lötjönen, J., Glocker, B., Tenovuo, O., and Rueckert, D. (2017). Regional brain morphometry in patients with traumatic brain injury based on acute- and chronic-phase magnetic resonance imaging. PLoS One 12, e0188152. 29182625 10.1371/journal.pone.0188152 PMC5705131

[42] Bigler, E.D. (2013). Traumatic brain injury, neuroimaging, and neurodegeneration. Front. Hum. Neurosci. 7, 395. 23964217 10.3389/fnhum.2013.00395 PMC3734373

[43] Jarrett, M., Tam, R., Hernández-Torres, E., Martin, N., Perera, W., Zhao, Y., Shahinfard, E., Dadachanji, S., Taunton, J., Li, D.K., and Rauscher, A. (2016). A prospective pilot investigation of brain volume, white matter hyperintensities, and hemorrhagic lesions after mild traumatic brain injury. Front. Neurol. 7, 11. 26903944 10.3389/fneur.2016.00011 PMC4751255

[44] Mori, S., Oishi, K., Jiang, H., Jiang, L., Li, X., Akhter, K., Hua, K., Faria, A.V., Mahmood, A., Woods, R., Toga, A.W., Pike, G.B., Neto, P.R., Evans, A., Zhang, J., Huang, H., Miller, M.I., van Zijl, P., and Mazziotta, J. (2008). Stereotaxic white matter atlas based on diffusion tensor imaging in an ICBM template. NeuroImage 40, 570–582. 18255316 10.1016/j.neuroimage.2007.12.035 PMC2478641

[45] Vrenken, H., Vos, E., Flier, W., Sluimer, I., Cover, K., Knol, D., and Barkhof, F. (2014). Validation of the automated method VIENA: an accurate, precise, and robust measure of ventricular enlargement. Hum. Brain Mapp. 35, 1101–1110. 23362163 10.1002/hbm.22237 PMC6869396

[46] Shen, X., Tokoglu, F., Papademetris, X., and Constable, R.T. (2013). Groupwise whole-brain parcellation from resting-state fMRI data for network node identification. NeuroImage, 82, 403–415. 23747961 10.1016/j.neuroimage.2013.05.081 PMC3759540

[47] McCuen, E., Svaldi, D., Breedlove, K., Kraz, N., Cummiskey, B., Breedlove, E., Traver, J., Desmond, K., Hannemann, R., Zanath, E., Guerra, A., Leverenz, L., Talavage, T., and Nauman, E. (2015). Collegiate women's soccer players suffer greater cumulative head impacts than their high school counterparts. J. Biomech. 48, 3720–3723. 26329462 10.1016/j.jbiomech.2015.08.003

[48] Fonov, V.S., Evans, A.C., McKinstry, R.C., Almli, C.R., and Collins, D.L. (2009). Unbiased nonlinear average age-appropriate brain templates from birth to adulthood. NeuroImage 47, Suppl. 1, S102.

[49] Cox, R.W. (1996). AFNI: software for analysis and visualization of functional magnetic resonance neuroimages. Comput. Biomed. Res. 29, 162–173. 8812068 10.1006/cbmr.1996.0014

[50] Broglio, S.P., Schnebel, B., Sosnoff, J.J., Shin, S., Fend, X., He, X., and Zimmerman, J. (2010). Biomechanical properties of concussions in high school football. Med. Sci. Sports Exerc. 42, 2064–2071. 20351593 10.1249/MSS.0b013e3181dd9156 PMC2943536

[51] Cummiskey, B., Schiffmiller, D., Talavage, T.M., Leverenz, L., Meyer, J.J., Adams, D., and Nauman, E.A. (2017). Reliability and accuracy of helmet-mounted and head-mounted devices used to measure head accelerations. Proc. Inst. Mech. Eng. P J. Sports Eng. Technol. 231, 144–153.

[52] Glantz, S.A., Slinker, B.K., and Neilands, T.B. (eds). (2016). *Primer of Applied Regression and Analysis of Variance*, 3rd ed. McGraw Hill Education: New York.

[53] Mu, S.H., Xu, M., Duan, J.X., Zhang, J., and Tan, L.H. (2017). Localizing age-related changes in brain structure using voxel-based morphometry. Neural Plast. 2017, 6303512. 28194282 10.1155/2017/6303512 PMC5282440

[54] Lenroot, R.K., Gogtay, N., Greenstein, D.K., Wells, E.M., Wallace, G.L., Clasen, L.S., Blumenthal, J.D., Lerch, J., Zijdenbos, A.P., Evans, A.C., Thompson, P.M., and Giedd, J.N. (2007). Sexual dimorphism of brain developmental trajectories during childhood and adolescence. NeuroImage 36, 1065–1073. 17513132 10.1016/j.neuroimage.2007.03.053 PMC2040300

[55] Ge, Y., Grossman, R.I., Babb, J.S., Rabin, M.L., Mannon, L.J., and Kolson, D.L. (2002). Age-related total gray matter and white matter changes in normal adult brain. Part I: volumetric MR imaging analysis. Am. J. Neuroradiol. 23, 1327–1333. 12223373 PMC7976241

[56] Ji, S., Ghadyani, H., Bolander, R.P., Beckwith, J.G., Ford, J.C., McAllister, T.W., Flashman, L.A., Paulsen, K.D., Ernstrom, K., Jain, S., Raman, R., Zhang, L., and Greenwald, R.M. (2014). Parametric comparisons of intracranial mechanical responses from three validated finite element models of the human head. Ann. Biomed. Eng. 42, 11–24. 24077860 10.1007/s10439-013-0907-2 PMC4397967

[57] Gurdjian, E.S. (1976). Cerebral contusions: re-evaluation of the mechanism of their development. J. Trauma 16, 35–51. 1246097

[58] Lee, T., Lycke, R., Auger, J., Music, J., Dziekan, M., Newman, S., Talavage, T., Leverenz, L., and Nauman, E. (2021). Head acceleration event metrics in youth contact sports more dependent on sport than level of play. Proc. Inst. Mech. Eng. H 235, 208–221. 33183139 10.1177/0954411920970812

[59] Poole, V.N., Breedlove, E.L., Shenk, T.E., Abbas, K., Robinson, M.E., Leverenz, L.J., and Talavage, T.M. (2015). Sub-concussive hit characteristics predict deviant brain metabolism in football athletes. Dev. Neuropsychol. 40, 12–17. 25649774 10.1080/87565641.2014.984810

[60] Svaldi, D.O., McCuen, E.C., Joshi, C., Robinson, M.E., Nho, Y., Hannemann, R., Nauman, E.A., Leverenz, L.J., and Talavage, T.M. (2017). Cerebrovascular reactivity changes in asymptomatic female athletes attributable to high school soccer participation. Brain Imaging Behav. 11, 98–112. 26809358 10.1007/s11682-016-9509-6

[61] Tremblay, S., Desjardins, M., Bermudez, P., Iturria-Medina, Y., Evans, A.C., Jolicoeur, P., and De Beaumont, L. (2019). Mild traumatic brain injury: the effect of age at trauma onset on brain structure integrity. Neuroimage Clin. 23, 101907. 31233955 10.1016/j.nicl.2019.101907 PMC6595074

[62] Smits, M., Houston, G.C., Dippel, D.W., Wielopolski, P.A., Vernooij, M.W., Koudstaal, P.J., Hunink, M.G., and van der Lugt, A. (2011). Microstructural brain injury in post-concussion syndrome after minor head injury. Neuroradiology 53, 553–563. 20924757 10.1007/s00234-010-0774-6 PMC3139069

[63] Mouzon, B.C., Bachmeier, C., Ferro, A., Ojo, J.O., Crynen, G., Acker, C.M., Davies, P., Mullan, M., Stewart, W., and Crawford, F. (2014). Chronic neuropathological and neurobehavioral changes in a repetitive mild traumatic brain injury model. Ann. Neurol. 75, 241–254. 24243523 10.1002/ana.24064

[64] Holmin, S., Mathiesen, T., and Shetye, J. (1995). Intracerebral inflammatory response to experimental brain contusion. Acta Neurochir. 132, 110–119 7754844 10.1007/BF01404857

[65] Holmin, S., Soderlund, J., and Biberfeld, P. (1998). Intracerebral inflammation after human brain contusion. Neurosurgery 42, 291–298. 9482179 10.1097/00006123-199802000-00047

[66] Unterberg, A.W., Stover, J., Kress, B., and Kiening, K.L. (2004). Edema and brain trauma. Neuroscience 129, 1021–1029. 15561417 10.1016/j.neuroscience.2004.06.046

[67] Conti, A., Raghupathi, R., Trojanowski, J., and Mcintosh, T. (1998). Experimental brain injury induces regionally distinct apoptosis in the acute and delayed post-traumatic period. J. Neurosci. 18, 5663–5672. 9671657 10.1523/JNEUROSCI.18-15-05663.1998 PMC6793063

[68] Wong, J., Hoe, N.W., and Zhiwei, F. (2005) Apoptosis and traumatic brain injury. Neurocrit. Care 3, 177–182. 16174891 10.1385/NCC:3:2:177

[69] Christensen, J., Wright, D.K., and Yamakawa, G.R. (2020). Repetitive mild traumatic brain injury alters glymphatic clearance rates in limbic structures of adolescent female rats. Sci. Rep. 10, 6254. 32277097 10.1038/s41598-020-63022-7 PMC7148360

[70] Plog, B.A., Dashnaw, M.L., Hitomi, E., Peng, W., Liao, Y., Lou, N., Deane, R., and Nedergaard, M. (2015). Biomarkers of traumatic injury are transported from brain to blood via the glymphatic system. J. Neurosci. 35, 518–526. 25589747 10.1523/JNEUROSCI.3742-14.2015 PMC4293408

[71] Boto, J., Gkinis, G., and Roche, A. (2017). Evaluating anorexia-related brain atrophy using MP2RAGE-based morphometry. Radiology 27, 5064–5072 10.1007/s00330-017-4914-9 28639048

[72] Seitz, J., Walter, M., Mainz, V., Herpertz-Dahlmann, B., Konrad, K., and von Polier, G. (2015). Brain volume reduction predicts weight development in adolescent patients with anorexia nervosa. J. Psychiatr. Res. 68, 228–237. 26228424 10.1016/j.jpsychires.2015.06.019

[73] Boghi, A., Sterpone, S., Sales, S., D'Agata, F., Bradac, G.B., Zullo, G., and Munno, D. (2011). In vivo evidence of global and focal brain alterations in anorexia nervosa. Psychiatry Res. 192, 154–159. 21546219 10.1016/j.pscychresns.2010.12.008

[74] Blasel, S., Pilatus, U., Magerkurth, J., Stauffenberg, M., Vronski, D., Mueller, M., Woeckel, L., and Hattingen, E. (2012). Metabolic gray matter changes of adolescents with anorexia nervosa in combined MR proton and phosphorus spectroscopy. Neuroradiology 54, 753–764. 22210349 10.1007/s00234-011-1001-9

[75] Biller, A., Reuter, M., Patenaude, B., Homola, G.A., Breuer, F., Bendszus, M., and Bartsch, A.J. (2015). Responses of the human brain to mild dehydration and rehydration explored in vivo by 1H-MR imaging and spectroscopy. Am. J. Neuroradiol.. 36, 2277–2284. 26381562 10.3174/ajnr.A4508 PMC4916775

[76] Kempton, M.J., Ettinger, U., Foster, R., Williams, S.C., Calvert, G.A., Hampshire, A., Zelaya, F.O., O'Gorman, R.L., McMorris, T., Owen, A.M., and Smith, M.S. (2011). Dehydration affects brain structure and function in healthy adolescents. Hum. Brain Mapp. 32, 71–79. 20336685 10.1002/hbm.20999 PMC6869970

[77] Fehily, B., and Fitzgerald, M. (2017). Repeated mild traumatic brain injury: potential mechanisms of damage. Cell Transplant. 26, 1131–1155. 28933213 10.1177/0963689717714092 PMC5657727

[78] Chen, J., He, W., and Hu, X. (2017). A role for ErbB signaling in the induction of reactive astrogliosis. Cell Discov. 3, 17044. 29238610 10.1038/celldisc.2017.44 PMC5717352

[79] Loane, D.J., Kumar, A., Stoica, B.A., Cabatbat, R., and Faden, A.I. (2014). Progressive neurodegeneration after experimental brain trauma: association with chronic microglial activation. J. Neuropathol. Exp. Neurol. 73, 14–29. 24335533 10.1097/NEN.0000000000000021 PMC4267248

[80] Frintrop, L., Trinh, S., Liesbrock, J., Leunissen, C., Kempermann, J., Etdöger, S., Kas, M.J., Tolba, R., Heussen, N., Neulen, J., Konrad, K., Päfgen, V., Kiessling, F., Herpertz-Dahlmann, B., Beyer, C., and Seitz, J. (2019). The reduction of astrocytes and brain volume loss in anorexia nervosa-the impact of starvation and refeeding in a rodent model. Transl. Psychiatry 9, 159. 31164627 10.1038/s41398-019-0493-7 PMC6548775

[81] Reyes-Haro, D., Labrada-Moncada, F.E., Varman, D.R., Krüger, J., Morales, T., Miledi, R., and Martínez-Torres, A. (2016). Anorexia reduces GFAP+ cell density in the rat hippocampus. Neural Plast. 2016, 2426413. 27579183 10.1155/2016/2426413 PMC4992534

[82] Harris, J.L., Choi, I.Y., and Brooks, W.M. (2015). Probing astrocyte metabolism in vivo: proton magnetic resonance spectroscopy in the injured and aging brain. Front. Aging Neurosci. 7, 202. 26578948 10.3389/fnagi.2015.00202 PMC4623195

[83] Redell, J.B., Maynard, M.E., Underwood, E.L., Vita, S.M., Dash, P.K., and Kobori, N. (2020). Traumatic brain injury and hippocampal neurogenesis: Functional implications. Exp. Neurol. 331, 113372. 32504636 10.1016/j.expneurol.2020.113372 PMC7803458

[84] Diotel, N., Lübke, L., Strähle, U., and Rastegar, S. (2020). Common and distinct features of adult neurogenesis and regeneration in the telencephalon of zebrafish and mammals. Front. Neurosci. 14, 568930. 33071740 10.3389/fnins.2020.568930 PMC7538694

[85] Rodríguez, J.J., and Verkhratsky, A. (2011). Neurogenesis in Alzheimer's disease. J. Anat. 219, 78–89. 21323664 10.1111/j.1469-7580.2011.01343.x PMC3130162

[86] Rinholm, J., Vervaeke, K., Tadross, M., Tkachuk, A., Kopek, B., Brown, T., Bergersen, L., and Clayton, D. (2016). Movement and structure of mitochondria in oligodendrocytes and their myelin sheaths. Glia 64, 810–825. 26775288 10.1002/glia.22965

[87] Lindfors, C., Nilsson, I.A., Garcia-Roves, P.M., Zuberi, A.R., Karimi, M., Donahue, L.R., Roopenian, D.C., Mulder, J., Uhlén, M., Ekström, T.J., Davisson, M.T., Hökfelt, T.G., Schalling, M., and Johansen, J.E. (2011). Hypothalamic mitochondrial dysfunction associated with anorexia in the anx/anx mouse. Proc. Natl. Acad. Sci. U. S. A. 108, 18108–18113. 22025706 10.1073/pnas.1114863108 PMC3207677

[88] Blasel, S., Pilatus, U., and Magerkurth, J. (2012). Metabolic gray matter changes of adolescents with anorexia nervosa in combined MR proton and phosphorus spectroscopy. Neuroradiology 54, 753–764. 22210349 10.1007/s00234-011-1001-9

